# Structurally restricted message-passing within shallow architectures for explainable network-level brain decoding on small cohorts

**DOI:** 10.3389/fnbeh.2026.1833995

**Published:** 2026-09-23

**Authors:** José Diogo Marques dos Santos, Maria Beatriz Ramos, Luís Paulo Reis, José Paulo Marques dos Santos, Bruno Direito

**Affiliations:** 1 Faculty of Engineering, University of Porto, Porto, Portugal; 2 LIACC – Artificial Intelligence and Computer Science Laboratory, University of Porto, Porto, Portugal; 3 Faculty of Medicine, University of Porto, Porto, Portugal; 4 LASI – Intelligent Systems Associate Laboratory, Guimarães, Portugal; 5 University of Maia, Maia, Portugal; 6 CISUC – Centre for Informatics and Systems, University of Coimbra, Coimbra, Portugal

**Keywords:** diffusion tensor imaging, explainable artificial intelligence, fMRI, graph neural networks, message-passing, shallow neural networks, structural connectivity, theory of mind

## Abstract

**Introduction:**

Applying artificial intelligence to functional magnetic resonance imaging (fMRI) has advanced the modelling of neural activity, but deep architectures such as graph neural networks (GNNs) require large samples, limiting their use in the small cohorts typical of task-based fMRI. Shallow neural networks (SNNs) are robust with few samples but treat brain regions independently, overlooking the brain’s network organization. We propose a structurally constrained message-passing framework that integrates diffusion-based structural connectivity, from a previously published group connectome, with region-level fMRI signals in a shallow architecture.

**Methods:**

The task is a binary classification of individual video trials as mentalizing or random, decoded from the fMRI response, in a Theory of Mind paradigm from the Human Connectome Project Young Adult dataset (30 subjects) under subject-wise 3-fold cross-validation. Two shallow classifiers are compared on the same data: the baseline classifies each trial from the regional fMRI signals as independent inputs, whereas the proposed model first applies one message-passing step, replacing each region’s signal with a structurally connected combination of its neighbors’ signals, so that classification operates at the level of anatomically defined networks. Interpretability is assessed using SHAP (SHapley Additive exPlanations).

**Results:**

Across folds, the proposed model matches the baseline (mean retrained accuracy 85.1%; 84.7% on the shared partition), the accuracy lost to pruning being recovered by retraining in every fold. The magnitude of the inputs’ contributions is reproducible across folds, whereas their direction is not. Only about a quarter of the consistently retained inputs also preserve their sign, so a cross-fold sign-consistency criterion is applied to retain reliable explanations. This identifies seven networks contributing robustly to the mentalizing class, located in the temporo-parietal junction, inferior parietal, and medial temporal cortices and overlapping a literature-based mentalizing map (overlap coefficient 0.383). Within these networks, Cohen’s D ranks the relative signal magnitude of the constituent regions.

**Discussion:**

Overall, structural constraints improve biological interpretability without compromising classification performance, while cross-validation establishes which explanations are stable, offering a compact and interpretable approach to network-level brain decoding in small cohorts and a basis that may be applied to connectivity disorders.

## 1 Introduction

Understanding how the brain produces sophisticated cognitive and social behavior remains a major issue in neuroscience. Functional magnetic resonance imaging (fMRI) has revolutionized the mapping of neural activity. However, most machine learning approaches to fMRI data fail to account for the anatomical pathways that connect brain regions and treat their data as isolated entities (Mohammadi and Karwowski, 2025). This is a serious shortcoming, as there is converging evidence that considers a range of neurological and psychiatric diseases, from Alzheimer’s disease to schizophrenia, as products of large network connectivity-related syndromes instead of isolated regional dysfunction (Bassett and Sporns, 2017; Du et al., 2018; Repple et al., 2023). In parallel, the opacity of artificial neural network classifiers remains a problem in their translation into neuroscientific knowledge (Farahani et al., 2022). Additionally, introducing graph neural networks (GNNs) to model the brain’s non-Euclidean nature requires large amounts of data for proper training, which is only available in extensive and costly studies. Addressing these gaps requires developing analytical frameworks that are simultaneously constrained by brain anatomy yet capable of providing principled explanations in a frugal data frame. The present study introduces a framework that combines diffusion-based structural connectivity into a shallow neural network classifier using message-passing, while analyzing its classifications using Explainable Artificial Intelligence approaches within the context of a Theory of Mind approach.

Artificial neural networks (ANNs) can extract meaningful patterns from complex datasets, producing models with high predictive accuracy when properly trained and good generalization when the input data are sufficiently representative (Haykin, 2009). In parallel, Functional Magnetic Resonance Imaging (fMRI) has become one of the most widely used techniques for studying the functioning human brain, producing four-dimensional maps of neural activity across space and time. Applications of fMRI range from investigating mental disorders to brain-decoding approaches aimed at understanding cognitive processes. Early reviews of machine learning methods for fMRI classification highlighted important methodological concerns regarding the use of ANNs in this context (Pereira et al., 2009). In particular, two key issues were emphasized: (1) whether the improvement in classification performance justifies the increased complexity of ANN models, and (2) the “black box” nature of such models, which complicates their explanation and interpretation.

Furthermore, in an opinion article, Wen et al. (2018) discuss the use of diverse ANN architectures for diagnosing cognitive impairments from fMRI data. Although they highlight the potential of deep models to advance mental health research, they emphasize a critical limitation: typical fMRI datasets are relatively small, increasing the risk of overfitting in complex Deep Neural Networks (DNNs). These constraints necessitate architectures that balance predictive performance and robustness in low-data regimes. Nevertheless, ANNs remain highly relevant in Social Neuroscience, where they are used to (i) predict behavior from brain activity, (ii) quantify naturalistic social stimuli and interactions, and (iii) generate cognitive models of social brain function (Sievers and Thornton, 2024).

More recent work has begun to address these concerns by developing procedures specifically tailored to the characteristics of fMRI data. Such approaches have demonstrated that ANN-based models can produce results consistent with established findings in Neuroscience, including in cognitive paradigms related to Theory of Mind (ToM) (Marques dos Santos and Marques dos Santos, 2022, 2023; Marques dos Santos et al., 2025). However, these procedures have primarily focused on region-level analyses in which each brain region is treated independently. As a result, they identify which regions contribute to classification but do not address how these regions interact as components of anatomically organized brain networks.

This limitation is particularly relevant in the context of neurological and psychiatric disorders. Increasing evidence suggests that many such conditions are better understood as disorders of large-scale network dysfunction rather than isolated regional abnormalities. For example, Alzheimer’s disease (AD), Mild Cognitive Impairment (MCI), Attention Deficit Hyperactivity Disorder (ADHD), Autism Spectrum Disorder (ASD), Bipolar Disorder, and Schizophrenia have increasingly been conceptualized as dysconnectivity syndromes involving disruptions of large-scale brain networks (Du et al., 2018). Consequently, understanding not only which brain regions are engaged but also how they interact within biologically plausible networks is essential for advancing translational applications. More broadly, advances in artificial intelligence applied to medical imaging have been highlighted as potentially contributing to global health initiatives such as United Nations Sustainable Development Goal 3, which aims to ensure good health and well-being for people of all ages (Chauhan et al., 2024). By improving the analysis and interpretation of neuroimaging data, ANN-based methods may, therefore, contribute to both fundamental neuroscience research and clinical innovation. Importantly, the usefulness of such models is likely to increase as their design more closely reflects the brain’s biological organization.

To address the limitations of region-level analysis, the present study integrates diffusion-based structural connectivity derived from Diffusion Tensor Imaging (DTI) into a shallow neural network (SNN) classifier through a message-passing mechanism. Message-passing, commonly used in graph-based architectures, enables information to propagate along anatomically defined pathways. In the proposed framework, this mechanism allows signals from individual brain regions to be combined according to structural connectivity patterns. Importantly, it is implemented within a shallow architecture designed to perform reliably under the relatively small sample sizes typical of task-based fMRI studies. This design enables classification at the level of structurally defined networks while preserving the stability advantages associated with SNNs.

Although there has been work exploring the simplification of GNNs, with the Simplified Graph Convolutional Network (SGC) (Wu et al., 2019) being the most apparent analogue, our approach differs from SGC in motivation, structure, and the role of the graph. SGC starts from a deep architecture and simplifies it. On the other hand, the structural connectome is an extremely sparse graph: most pairs of cortical regions are not directly connected, a regime in which conventional message-passing GNNs propagate information slowly and tend to underperform regardless of architectural depth. Furthermore, the low-data regime characteristic of task-based fMRI precludes the use of deep GNN architectures regardless of their theoretical merits at scale. Accordingly, a single propagation step is used, parameterized by a fixed, binary anatomical adjacency derived from DTI tractography, not learned from the functional data, and its output is fed to a non-linear shallow classifier rather than to a linear one. Two structural differences follow. The graph operation and the classifier are decoupled here, whereas in SGC the graph operation is subsumed into the classifier as the linear model after collapse; the same propagation step can therefore in principle precede any downstream architecture. The normalization choices also differ: SGC uses symmetric Laplacian normalization on the (typically dense) graph, whereas column-sum (degree) normalization is applied here on a binary, sparse anatomical adjacency.

To address the second concern raised above, the “black box” nature of ANN models, the proposed framework is combined with interpretability and explainability methods. Even in relatively compact models such as the present SNN, which contains 3,620 connections, the network’s internal operations are too complex to be readily interpreted by direct inspection. Explainable Artificial Intelligence (XAI) aims to overcome this challenge by providing tools that clarify how machine learning models reach their decisions. According to Doran et al. (2018), four key challenges characterize explainability in dense ANN models: opacity, where the internal mechanisms are inaccessible to users; interpretability, where the mechanisms can be analyzed mathematically; comprehensibility, where symbolic outputs enable human-understandable explanations; and full explainability, where automated reasoning generates self-contained explanations. XAI methods may be applied to increase the transparency of ANN models, enabling knowledge extraction from fMRI data in the context of a typical cognitive task (Roscher et al., 2020).

The cognitive paradigm investigated in this study originates from the field of Social Neuroscience, which examines the neural bases of social cognition. Over the past two decades, advances in fMRI have greatly accelerated research in this domain (Adolphs, 2001; Decety and Keenan, 2006; Norman et al., 2010; Saxe, 2006). Among the most extensively studied constructs in social neuroscience is the Theory of Mind, also referred to as cognitive empathy or mentalizing (Shamay-Tsoory et al., 2009). This concept describes the human ability to attribute beliefs, intentions, and goals to others during social interactions (Singer and Tusche, 2014). Such attribution of mental states plays a central role in successful social behavior. Although the extent to which ToM abilities are uniquely human remains debated (Call and Tomasello, 2008; Premack and Woodruff, 1978; van Mourik et al., 2024; Vilone and Longo, 2021), the concept remains fundamental for understanding the neural mechanisms underlying social cognition. The data used in the present study were acquired using a Theory of Mind paradigm that replicates the classic experiment introduced by Fritz Heider and Marianne Simmel in 1944 (Heider and Simmel, 1944), in which participants inferred social interactions from the movement of simple geometric shapes.

In sum, the present study aims to further develop on previous region-level approaches by investigating whether incorporating structural connectivity into an ANN framework can enable biologically meaningful network-level brain decoding while preserving classification performance. Specifically, diffusion-based connectivity is integrated into a shallow neural network via a message-passing mechanism, enabling signals from individual regions to be combined along anatomically plausible pathways. This network-level representation is then examined using methods from Explainable Artificial Intelligence to determine which structurally defined subnetworks contribute to classification in a Theory-of-Mind paradigm. In doing so, the study addresses several key questions: whether structurally constrained models can achieve predictive performance comparable to region-based approaches, whether such models yield explanations consistent with established findings in Social Neuroscience, whether integrating anatomical connectivity can improve the biological interpretability of ANN-based analyses of Functional Magnetic Resonance Imaging data, and whether the resulting performance and explanations remain stable across cross-validation folds. Together, these objectives aim to evaluate whether combining structural constraints with interpretable machine learning provides a viable framework for studying both cognitive processes and network-level brain organization. Thus, our hypothesis can be summarized as: The inclusion of the message-passing mechanism yields a more biologically accurate and network-level analysis than the region-level classifier, without hampering model performance.

## 2 Methods

### 2.1 Dataset

The dataset comprised the social paradigm of 30 subjects from the Human Connectome Project Young Adult Dataset,^1^ specifically subjects 100307 to 124422 from the 100 Unrelated Subjects subset (Elam et al., 2021; Van Essen and Glasser, 2016; Van Essen et al., 2013). The task consists of 20-s videos showing moving geometric shapes, whose motion is either random (rnd) or depicts interactions that require mentalizing (mnt) (Heider and Simmel, 1944). Participants classify each video as random (rnd), mentalizing (mnt), or unsure, or may provide no response. The videos are presented in their entirety regardless of when a response is given. Only correctly classified trials are included in the analysis. Each participant completes two runs with different scanner phase-encoding directions: left-to-right (LR) and right-to-left (RL). Each run includes five stimuli (two from one condition and three from the other, counterbalanced across runs). Consequently, each participant evaluates five stimuli per condition, resulting in a total of 10 stimuli. Figure 1 shows the stimuli sequence of the paradigm.

**Figure 1:**
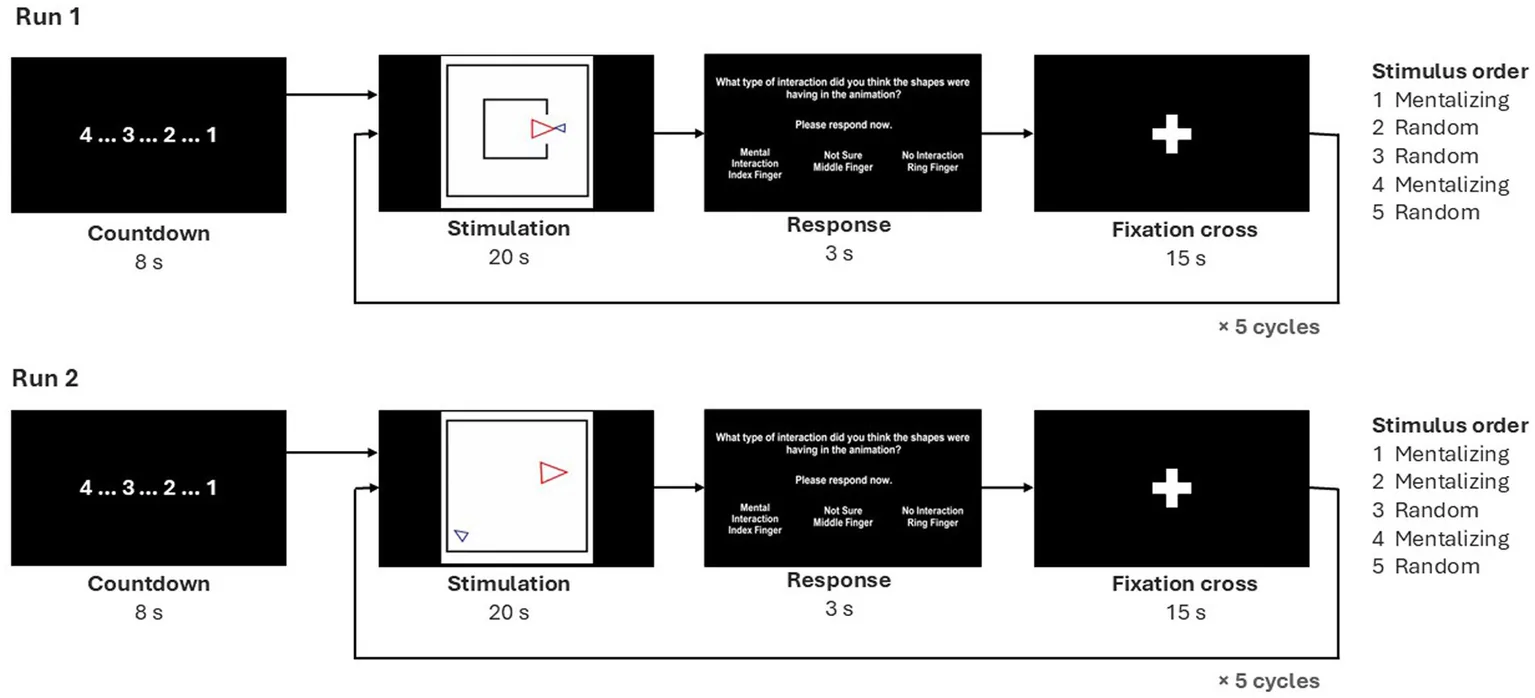
Schematic of HCP’s social paradigm stimuli sequence. The sequence starts by a countdown from 4 to 1, each number is shown for 2 seconds for a total of 8 seconds. Each stimulation movie is 20 seconds long and has movement of geometric figures. The change between mentalizing (mnt) and random (rnd) are whether the movement is interactions between the shapes or random movement. After each stimulation movie there is a 3 second response slide, followed by a 15 second fixation cross between stimulations.

### 2.2 Feature extraction

For SNN feature extraction, the brain is parcellated using the Human Connectome Project Multimodal Parcellation Atlas (HCP-MMP1.0) (Glasser et al., 2016). Each subject’s BOLD (blood oxygen level-dependent) image is masked with the 360 regions defined in the atlas, and the voxels’ time series within each region are averaged, yielding 360 regional mean time series per session. The complete list of all 360 regions, including their IDs, names, and locations, is provided in Supplementary Table 1. The time series are segmented according to the stimulus sequence, and the 7th to 9th time points after stimulus onset are averaged to define a single feature per region for each instance. The time points are chosen based on a canonical double-gamma Hemodynamic Response Function (HRF) that models the signal lag relative to brain activation. As the TR is 0.72 s, the 7th through 9th time points correspond between 5.04 and 6.48 s after stimulus onset. Input matrices are then constructed and standardized. The training and test matrices are standardized independently: each is centered and scaled using its own grand mean and grand standard deviation, computed over the entire matrix (all regions and instances), so that the standardization of one set does not depend on the other. Standardization is applied to the region-level signals, prior to the message-passing step.

### 2.3 Model architecture

#### 2.3.1 Structural connectivity matrix

Structural connectivity is derived from a previously published HCP-MMP1.0-based group connectome (Baker et al., 2018; Briggs et al., 2018a; Briggs et al., 2018b; Briggs et al., 2018c; Briggs et al., 2018d; Briggs et al., 2018e; Conner et al., 2018a; Conner et al., 2018b; Conner et al., 2018c; Sali et al., 2018), obtained through anatomy-driven deterministic tractography on diffusion-weighted images from 10 subjects using Generalized Q-Sampling Imaging (ratio = 1.25; angular threshold = 45°), with manual seeding of established anatomical pathways. Connectivity is defined by the presence of streamlines between regions, resulting in a 360 × 360 binary matrix containing 2,100 connections. A diagonal term is added to the matrix to preserve intra-regional contributions during message-passing. The complete structural connectivity matrix is available in Supplementary Table 2.

#### 2.3.2 Message-passing mechanism

The message-passing mechanism constitutes the functional basis of Graph Neural Networks (GNNs). It iteratively updates the representation of each node as a function of the representations of its neighboring (connected) nodes. However, GNNs are typically deep architectures that require large amounts of data to ensure convergence to a solution with acceptable performance. In the present study, only a single message-passing step is performed. Mathematically, this operation corresponds to multiplying the signal matrices (training and testing) by the structural connectivity matrix. Thus, the signal of each network corresponds to the sum of the signals from all structurally connected regions. This procedure is defined as:


$$Z=S\cdot (C+I_{360})\cdot D^{-1}$$

Where *S* is the signal matrix obtained during feature extraction (an *n* × 360 matrix, with *n* being the number of instances), *C* represents the binary anatomical adjacency (a 360 × 360 matrix), *I_360_* is the identity matrix added to introduce regions’ self-loops, *D* is the diagonal degree matrix of *C + I_360_* (degree includes the self-loop), and *Z* is the resulting transformed signal matrix (an *n* × 360 matrix). The self-loop addition preserves each region’s own signal during aggregation; the right-multiplication by *D^−1^* applies degree-based normalization, dividing each aggregated column by its degree (including the self-loop). This correction mitigates signal dilution, which may occur when only a subset of regions within a network is recruited. In such cases, summing activation-related signal peaks with baseline activity can reduce the contrast between the activated and non-activated states of the network.

The result of this operation is a matrix with the same shape as the input signal matrix (*n* × 360), rather than a smaller set of networks. The input signal matrix has one column per brain region, and the transformed matrix likewise has 360 columns. The operation does not change the number of columns, it changes what each column contains. Before message-passing, column *j* holds the signal of region *j* alone. After message-passing, column *j* holds the degree-normalized sum (i.e., the average signal) of region *j* together with all regions structurally connected to it, with region *j* itself retained through the self-loop introduced by the diagonal term. Because a single message-passing step is performed, this set comprises only the directly connected (1-hop) neighbors. This set, an anchor region plus its direct neighbors in the anatomical adjacency matrix, is what is termed a “network.” There are therefore 360 such networks, one anchored on each region and indexed by that anchor region. A network may comprise a single region, when its anchor has no structural connections in the matrix (for example, networks 327 and 292), or several regions, when the anchor is connected to others (for example, network 280 aggregates the signals of regions 280, 296, and 328). The networks are not disjoint: a region can belong to more than one network, since it can be connected to several anchor regions (for example, region 302 belongs to networks 335, 301, and 321). Each of these 360 network signals then serves as a single input node of the SNN classifier.

#### 2.3.3 SNN classifier

To ensure consistency with previous studies and, therefore, discuss comparable results, the fully connected SNN comprises 360 input nodes (one per network), 10 hidden nodes arranged in a single hidden layer, and 2 output nodes (one for each class in the paradigm design). The network architecture is implemented using the AMORE library (version 0.2–15) (Limas et al., 2014), R (version 4.5.1), and RStudio (version 2025.05.1.513). The activation function applied to the hidden layer is the hyperbolic tangent (“tansig”), which produces outputs in the range ]−1, 1[ and is centered at zero. The output layer uses the sigmoid activation function, which outputs values in the range ]0, 1[. Figure 2 illustrates a simplified schematic representation of the classifier architecture. The message-passing step occurs between the first two layers in the schema, with the second layer (the output of the message-passing mechanism) serving as the input to the SNN classifier.

**Figure 2:**
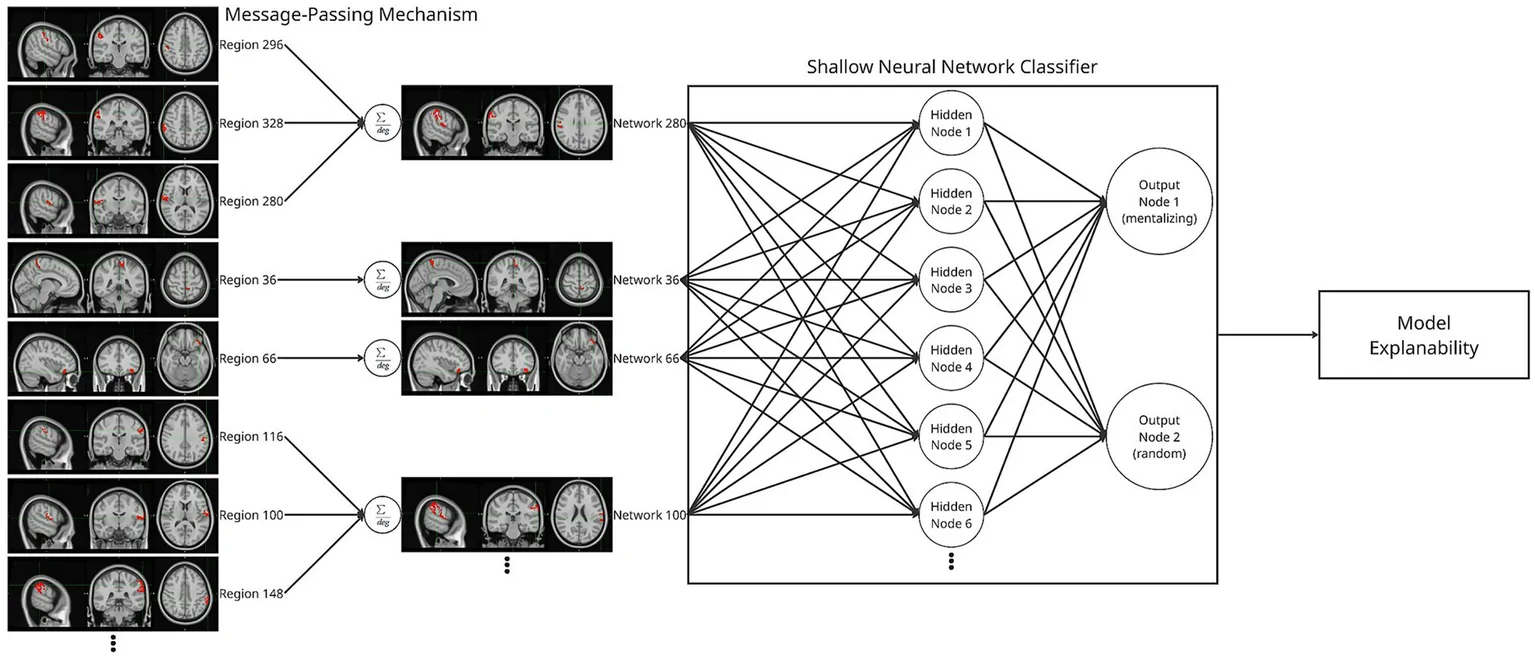
Message-passing mechanism on the SNN classifier. The connection between the first two layers (from regions to networks) is the message-passing mechanism. The following layers are the Shallow Neural Network classifier, and lastly, the model explainability steps are on the right.

### 2.4 Training, pruning, and retraining

Model performance is estimated using subject-wise 3-fold cross-validation. The 30 participants are partitioned into three disjoint folds of 10 subjects each. Partitioning is performed at the subject level, so that all instances from a given participant belong to the same fold, preventing leakage of subject-specific signal between training and testing; across the three folds, each subject is used for testing exactly once. For each fold, the two remaining folds (20 subjects) form the training set and the held-out fold (10 subjects) the test set. The held-out test sets comprise 85 instances (45 mnt, 40 rnd), 87 instances (48 mnt, 39 rnd), and 90 instances (54 mnt, 36 rnd) for the three folds respectively, totaling 262 instances (147 mnt, 115 rnd); each fold’s training set is formed by the instances of the remaining two folds. The message-passing operator is identical across folds, as it encodes a fixed anatomical prior rather than parameters learned from the functional data; only the classifier is refit. Within each fold, a grid search over the learning rate and momentum parameters is performed, with each parameter pair evaluated 100 times to account for variability due to random weight initialization. The best-performing pair is then used to train 50,000 networks differing only in their initial weights; the network with the highest accuracy is retained as that fold’s fully connected network. The first stage of the pruning procedure consists of ranking all possible path-weights. The concept of “path-weights” (Marques dos Santos and Marques dos Santos, 2022, 2023) refers to the product of the weights along a path from a given input node to a given output node through a single hidden node, as the present architecture includes only one hidden layer. Formally, a path-weight is defined as:


$$\text{path}-\text{weigh}t_{i,j,k}=w_{I_{i},H_{j}}\times w_{H_{j},O_{k}}$$

Where $w_{I_{i},H_{j}}$ is the weight between the input node, $I_{i}$ , and the hidden node, $H_{j}$ ; and $w_{H_{j},O_{k}}$ is the weight between the hidden node, $H_{j}$ , and the output node, $O_{k}$ . Therefore, $\text{path}-\text{weigh}t_{i,j,k}$ is the product of the weights found in the path from the input $I_{i}$ to output $O_{k}$ , passing by the hidden node, $H_{j}$ . A path-weight with a magnitude close to zero tends to attenuate the contribution of the input signal along that path and may therefore be pruned due to its low relevance. Conversely, a path-weight with a larger magnitude, whether positive or negative, exerts a stronger influence on classification and is therefore retained. The distribution of path-weights typically exhibits two elbow points, indicating that only a limited number of paths effectively transmit information from the input layer to influence the model output. The elbow points are computed using the “pathviewer” (R package) (Baliga et al., 2023). Path-weights lying between the positive and negative elbow points are pruned. This pruning is performed within each fold: the path-weight distribution and its two elbow points are determined separately for each fold’s fully connected network, so the pruning thresholds, the number of surviving paths, and the set of surviving networks are fold-specific. The intersection of the surviving inputs across folds is used in the subsequent explanation analysis. A more detailed description of this procedure is provided elsewhere (Marques dos Santos and Marques dos Santos, 2024). Figure 3 shows the positive and negative arms of this distribution of the ranked path-weights.

**Figure 3:**
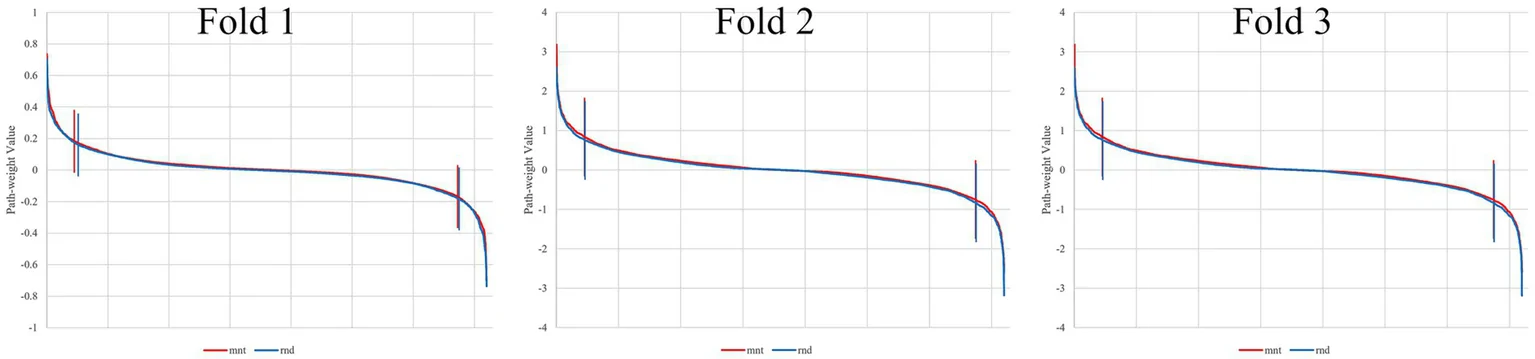
Path-weights of each fold plotted per output in decreasing order for each stimulus category. All plots have a tilde shape, meaning that most paths have little influence on the computation (because they are close to zero and tend to cancel the signal in the multiplication). Just a few have weights that move away from nullity. The latter are retained in the pruning process. Vertical lines signal the elbow points, which correspond to paths: mnt (red) 226 and 3,362, rnd (blue) 257 and 3,375 for fold 1; mnt (red) 226 and 3,371, rnd (blue) 230 and 3,375 for fold 2; mnt (red) 226 and 3,371, rnd (blue) 230 and 3,375 for fold 3. The paths that survive pruning are above the thresholds in the positive tail and below the thresholds in the negative tail of the distributions.

The resulting pruned network is then retrained to update the weights according to the new architecture. At this stage, a grid search is performed again to identify the best hyperparameters (learning rate and momentum). However, only one network is trained for each parameter pair, since the initial weights are deterministic and no longer randomly initialized. This step produces the retrained network. The training, pruning, and retraining procedure is carried out independently within each fold, producing three fully connected, three pruned, and three retrained networks.

### 2.5 Explaining

Shapley values are defined by:


$$\Phi_{A}=\sum_{S\subseteq F\backslash \{A\}}\frac{|S|!(|F|-|S|-1)!}{|F|!}[f(S\cup \{A\})-f(S)]$$

where *S* is the set of inputs to the SNN, with the exception of input *A*, which is the target of evaluation about its importance/contribution to the classification. The term [f(S∪{A})−f(S)] is essential for comprehending the significance of the Shapley values, as it compares two situations: the full set of inputs, i.e., f(S∪{A}) , and the situation where the targeted input is removed from the calculations, i.e., f(S) . Two groups of results may occur:

-
f(S∪{A})≈f(S)⇒[f(S∪{A})−f(S)]≅0 meaning that *A* has little importance/contribution to the classification;
-
[f(S∪{A})−f(S)]≫0 or [f(S∪{A})−f(S)]≪0 meaning that *A* is important for correct classification.

The computation of Shapley values is performed using the retrained network in Python with the SHAP library.^2^ SHAP (SHapley Additive exPlanations) provides a framework for interpreting model predictions by attributing contributions to individual features. Because the original network is implemented in R using the AMORE package, the model must first be translated to Python using PyTorch.^3^ To reproduce the original architecture, a custom activation function is implemented in Python to mimic the tansig activation used in AMORE. A network with the same architecture as the original model is first initialized in PyTorch, consisting of 360 input nodes, one hidden layer with 10 hidden nodes using the custom tansig activation function, and two output nodes with the sigmoid activation function. The network is initially created with randomly generated weights. The weights from the retrained network are then exported from R and imported into the PyTorch model in the appropriate format, replacing the randomly initialized weights. This procedure yields an exact replication of the network originally trained in R. The explanation stage is subsequently performed using the SHAP library. An explainer is created using the DeepExplainer function, which is based on an enhanced version of the DeepLIFT algorithm (Shrikumar et al., 2017). As shown in (Lundberg and Lee, 2017), this method approximates the conditional expectations of Shapley values by integrating over a large number of samples, such that the sum of the SHAP values equals the difference between the expected model output and the actual model prediction. The SHAP analysis uses these approximations of Shapley values to rank the features according to the change they produce in the model’s decision, thereby quantifying their importance in the SNN’s classification process. This procedure yields one value for each feature, for each instance, and for each class. These values can then be averaged in absolute magnitude to obtain a global ranking of feature importance. Furthermore, examining the relationship between the SHAP value and the corresponding input value reveals whether a feature has a positive or negative influence on the model’s decision. Specifically, this analysis indicates whether higher input values drive the prediction toward a given class or contribute to rejecting classification into that class.

Because performance is estimated by cross-validation, the explanation stage is repeated independently for each fold using that fold’s retrained network. Within each fold, SHAP values are computed for the held-out test instances using the DeepExplainer, with those same test instances serving as the background distribution, so that attributions are expressed relative to the mean prediction over the held-out fold. A fold-level importance is assigned to each surviving input by summing the absolute SHAP values over the mentalizing (mnt) instances only, so that the ranking reflects the inputs driving the mnt classification specifically.

Across folds, 93 inputs survive pruning in all three folds. These are ranked by their mean SHAP rank across folds, and the cross-fold concordance of the three rankings is quantified using Kendall’s coefficient of concordance (W). A sign-consistency criterion is then applied to isolate reliable mnt-related inputs: an input is retained only if it contributes positively to the mnt class in all three folds. Proceeding down the ranking, the seven highest-ranked mnt-positive inputs are each positive in all three folds, whereas the next mnt-positive input is positive in only two of the three folds and is therefore excluded as sign-inconsistent. This yields seven networks, which are carried forward to the regional analysis and to the spatial overlap comparison with the literature.

In this study, the SHAP analysis identifies the most important inputs (brain networks), i.e., ranks highly the brain networks whose putative absence would mostly impact a drop in accuracy. However, these must be compared to established neuroscientific knowledge. To establish the baseline for this comparison a GLM-based statistical parametric map of a similar mentalizing condition using Frith-Happé animations is extracted from the literature (Dureux et al., 2023). To obtain a quantitative measure of the overlap between this established baseline and the identified relevant networks from the SHAP analysis, the overlap coefficient is used. For this study, the overlap is mathematically given by:


$$\text{overlap}(X,Y)=\frac{|X\cap Y|}{\min(|X|,|Y|)}$$

Where overlap(X,Y) is the overlap coefficient between maps X and Y , ∣X∣ and ∣Y∣ are the total number of voxels in each map, and ∣X∩Y∣ is the number of voxels common to both maps. In practice, it provides a quantitative measure of how much of the identified networks also appear in the baseline GLM-based map.

### 2.6 Cohen’s D

Cohen’s D is an effect size measure based on differences between means. Effectively, it measures the distance between two sets of data points. Thus, Cohen’s D value closer to 0 means both sets of data points are close together, while a higher or lower (more negative) value means they are far apart. Mathematically, Cohen’s D is given by:


$$d=\frac{{\bar{x}}_{1}-{\bar{x}}_{2}}{s}$$

Where d is the Cohen’s D value, ${\bar{x}}_{1}$ is the mean of the first set of data points, ${\bar{x}}_{2}$ is the mean of the second set of data points, and s is the pooled standard deviation.

For each network selected by the cross-fold SHAP procedure described in the previous subsection, Cohen’s D is computed across its constituent regions; networks comprising a single region are excluded, as this comparison requires at least one region pair. Because Cohen’s D characterizes the extracted signal rather than the trained model, it is computed once over the full sample rather than per fold: the standardized n × 360 signal matrices (taken after standardization but before the message-passing step) of the held-out test subjects from the three folds are concatenated so that each subject contributes exactly once, reconstructing the complete set of 30 subjects, and only mentalizing (mnt) instances are retained. From this matrix, Cohen’s D is computed for every pair of regions, yielding a 360 × 360 matrix, from which the within-network submatrices of the selected networks are extracted. Computing the effect size on the pooled instances, rather than averaging per-fold estimates, avoids the upward bias that per-fold averaging introduces through the law of total variance and the positive small-sample bias of d within each fold.

## 3 Results

### 3.1 Region level classification (baseline)

First, the regional-level classification corresponds to the model reported in the literature (Marques dos Santos et al., 2025). It is trained on the same dataset and uses identical preprocessing and feature extraction procedures. Importantly, in the original study, the explanations produced by this analysis were examined and shown to be consistent with established findings in Neuroscience. Therefore, it provides a robust baseline against which the proposed message-passing mechanism can be compared. The performance of the three network configurations is summarized in Table 1, which reports confusion matrices, local precisions as well as both local and global accuracies for the fully connected, pruned, and retrained networks. The initial global accuracy is 88.2%, which decreases to 80.0% after pruning. Following retraining, the global accuracy increases to 84.7%. A detailed analysis of this model is provided in the original study.

**Table 1:** Confusion matrices, precisions, and accuracies of the fully connected, pruned, and retrained networks obtained for the region-level classifier in (Marques dos Santos et al., 2025).

Network	Stimulus		Prediction		Total
			mnt	rnd	
Fully Connected Network	Input	mnt	43	2	45
		rnd	8	32	40
	Total		51	34	85
	Accuracy (%)		95.6	80	88.2
	Precision (%)		84.3	94.1	
Pruned Network	Input	mnt	41	4	45
		rnd	13	27	40
	Total		54	31	85
	Accuracy (%)		91.1	67.5	80
	Precision (%)		75.9	87.1	
Retrained Network	Input	mnt	41	4	45
		rnd	9	31	40
	Total		50	35	85
	Accuracy (%)		91.1	77.5	84.7
	Precision (%)		82	77.5	

### 3.2 Network-level classification

The proposed network-level classifier is evaluated using subject-wise 3-fold cross-validation. Tables 2–4 report the confusion matrices, precisions, and accuracies for the fully connected, pruned, and retrained networks in folds 1, 2 and 3, respectively. The retrained global accuracies are 84.7, 88.5, and 82.2% across folds (mean 85.1%); the fully connected networks reach 87.1, 90.8, and 90.0% (mean 89.3%), and the pruned networks 77.6, 79.3, and 74.4% (mean 77.1%). On fold 1, whose partition is shared with the region-level baseline (Table 1), the retrained network-level model matches the region-level model exactly at the global level (both 84.7%), providing a comparison on identical test data.

**Table 2:** Confusion matrices, precisions, and accuracies of the fully connected, pruned, and retrained networks obtained for the fold 1 of the network-level classifier.

Network	Stimulus		Prediction		Total
			mnt	rnd	
Fully Connected Network	Input	mnt	43	2	45
		rnd	9	31	40
	Total		52	33	85
	Accuracy (%)		95.6	77.5	87.1
	Precision (%)		82.7	93.9	
Pruned Network	Input	mnt	38	7	45
		rnd	12	28	40
	Total		50	35	85
	Accuracy (%)		84.4	70	77.6
	Precision (%)		76	80	
Retrained Network	Input	mnt	43	2	45
		rnd	11	29	40
	Total		54	31	85
	Accuracy (%)		95.6	72.5	84.7
	Precision (%)		79.6	93.5	

**Table 3:** Confusion matrices, precisions, and accuracies of the fully connected, pruned, and retrained networks obtained for the fold 2 of the network-level classifier.

Network	Stimulus		Prediction		Total
			mnt	rnd	
Fully Connected Network	Input	mnt	43	5	48
		rnd	3	36	39
	Total		46	41	87
	Accuracy (%)		89.6	92.3	90.8
	Precision (%)		93.5	87.8	
Pruned Network	Input	mnt	40	8	48
		rnd	10	29	39
	Total		50	37	87
	Accuracy (%)		83.3	74.4	79.3
	Precision (%)		80	78.4	
Retrained Network	Input	mnt	44	4	48
		rnd	6	33	39
	Total		50	37	87
	Accuracy (%)		91.7	84.6	88.5
	Precision (%)		88	89.2	

**Table 4:** Confusion matrices, precisions, and accuracies of the fully connected, pruned, and retrained networks obtained for the fold 3 of the network-level classifier.

Network	Stimulus		Prediction		Total
			mnt	rnd	
Fully Connected Network	Input	mnt	48	6	54
		rnd	3	33	36
	Total		51	39	90
	Accuracy (%)		88.9	91.7	90.0
	Precision (%)		94.1	84.6	
Pruned Network	Input	mnt	45	9	54
		rnd	14	22	36
	Total		59	31	90
	Accuracy (%)		83.3	61.1	74.4
	Precision (%)		76.3	71.0	
Retrained Network	Input	mnt	45	9	54
		rnd	7	29	36
	Total		52	38	90
	Accuracy (%)		83.3	80.6	82.2
	Precision (%)		86.5	76.3	

The pattern observed in the single-split analysis is reproduced in all three folds: pruning reduces accuracy and retraining recovers most of the loss, returning global accuracy to a level comparable to the fully connected network. Local accuracy for the mnt class remains higher than for the rnd class in the retrained networks across folds (mean 90.2% vs. 79.2%), indicating that the model is more sensitive to mentalizing-related patterns.

### 3.3 SHAP values

In each fold, the SHAP analysis ranks the inputs by their mean absolute SHAP value, following the standard formulation. Figure 4 shows the beeswarm plots of the 20 highest-ranked inputs in each of the three folds. Because the task is a binary classification, this ordering is identical across the two classes, and, thus, only the mnt beeswarms are shown. These per-fold importance rankings are concordant (Kendall’s W = 0.61; permutation *p* < 0.001), indicating that the model relies on a consistent set of inputs irrespective of fold.

**Figure 4:**
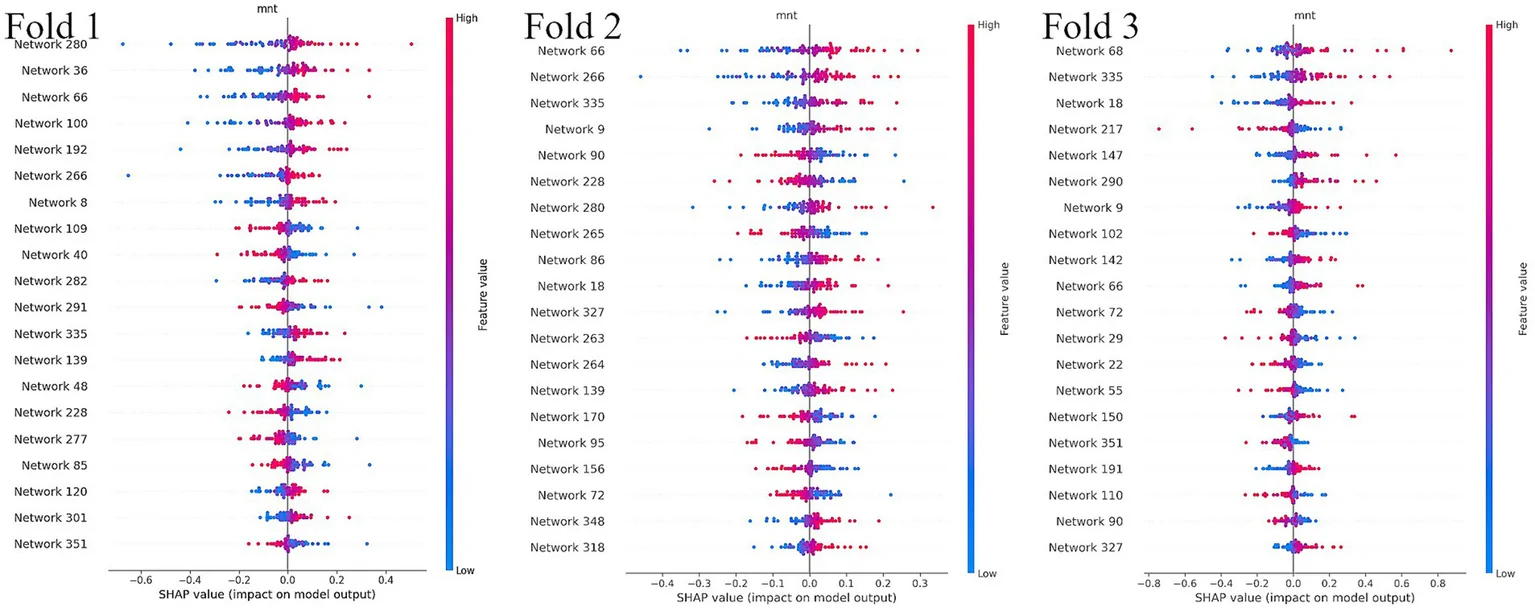
Summary plot containing the distribution of SHAP values of the retrained network for each of the twenty highest-ranked networks (inputs) for each fold. A higher rank means greater influence on the output computation, either positively (red on the right) or negatively (blue on the right). Since this is a binary classification only one of the classes is shown (mnt) as the plot for the other class (rnd) would be similar but with inverted polarities (positive and negative arms of the beeswarms change side).

While this confirms that the same inputs are repeatedly informative, it does not by itself establish which inputs are specifically and reliably associated with the mentalizing condition, because the absolute value discards the direction of each input’s contribution. To resolve this, the analysis is restricted to the 93 inputs that survive pruning in all three folds, and for each of these the signed SHAP values are summed over the mnt instances only, so that a positive value denotes a contribution toward the mnt class. Unlike the magnitude rankings, the resulting signed rankings are not concordant across folds (Kendall’s W = 0.37, which for three rankings is at the no-concordance level of W = 1/3; mean pairwise Spearman ρ̄ = 0.058; permutation *p* = 0.17). A diagnostic restricting the magnitude ranking to the same mnt instances confirms that this instability stems from the sign rather than from the reduced number of instances (magnitude, mnt instances: W = 0.57). Accordingly, only 24 of the 93 inputs (25.8%) preserve their contribution sign across all three folds, so a sign-consistency criterion is required to isolate reliable mnt-related inputs.

The 93 inputs are ranked by their mean rank across folds and screened for sign-consistency. The seven highest-ranked inputs each contribute positively to mnt in all three folds; the eighth (network 139) contributes positively in two folds but negatively in the third, breaking the sequence of sign-consistency, so the first seven inputs are retained. In decreasing order of importance these are networks 335, 327, 301, 280, 321, 100, and 292, with networks 335 and 327 the most highly ranked and stable. Table 5 lists these networks and their constituent regions. The full cross-fold ranking of all 93 inputs is provided in Supplementary Table 3.

**Table 5:** Brain networks and respective regions that consistently hold important information for the positive classification of the mentalizing class across folds.

Network	Region ID	Region name	Lobe	Cortex
335	302	Perirhinal_Ectorhinal_Cortex_R	Temp	Medial_Temporal
	335	ParaHippocampal_Area_2_R	Temp	Medial_Temporal
327	327	Area_PF_Opercular_R	Par	Inferior_Parietal
301	301	ProStriate_Area_R	Par	Posterior_Cingulate
	302	Perirhinal_Ectorhinal_Cortex_R	Temp	Medial_Temporal
280	280	Area_OP4-PV_R	Par	Posterior_Opercular
	296	Area_PFt_R	Par	Inferior_Parietal
	328	Area_PF_Complex_R	Par	Inferior_Parietal
321	302	Perirhinal_Ectorhinal_Cortex_R	Temp	Medial_Temporal
	315	Area_TF_R	Temp	Medial_Temporal
	321	Area_TemporoParietoOccipital_Junction_3_R	Par	Temporo-Parieto_Occipital_Junction
	352	Area_TG_Ventral_R	Temp	Lateral_Temporal
100	100	Area_OP4-PV_L	Par	Posterior_Opercular
	116	Area_PFt_L	Par	Inferior_Parietal
	148	Area_PF_Complex_L	Par	Inferior_Parietal
292	292	Anterior_Agranular_Insula_Complex_R	Ins	Insular_and_Frontal_Opercular

Their absence during Shapley values estimation leads to a significant drop in classification performance across folds.

The spatial expression of the seven networks is shown in Figure 5. Their correspondence with the mentalizing literature is assessed against the GLM-based statistical parametric map of a comparable mentalizing condition (Dureux et al., 2023). Because the BOLD signal originates primarily from gray matter and the HCP-MMP1.0 atlas comprises only cortical gray-matter parcels, the GLM map is masked with the MMP1.0 atlas, excluding white-matter voxels where task-related activation is not biologically expected and arises mainly from spatial-smoothing partial-volume effects. The seven networks span 3,328 voxels and the masked GLM map 17,875 voxels, with an intersection of 1,274 voxels, yielding an overlap coefficient of 0.383 (Figure 6); that is, 38.3% of the network voxels fall within the literature-based mentalizing map (Table 5).

**Figure 5:**
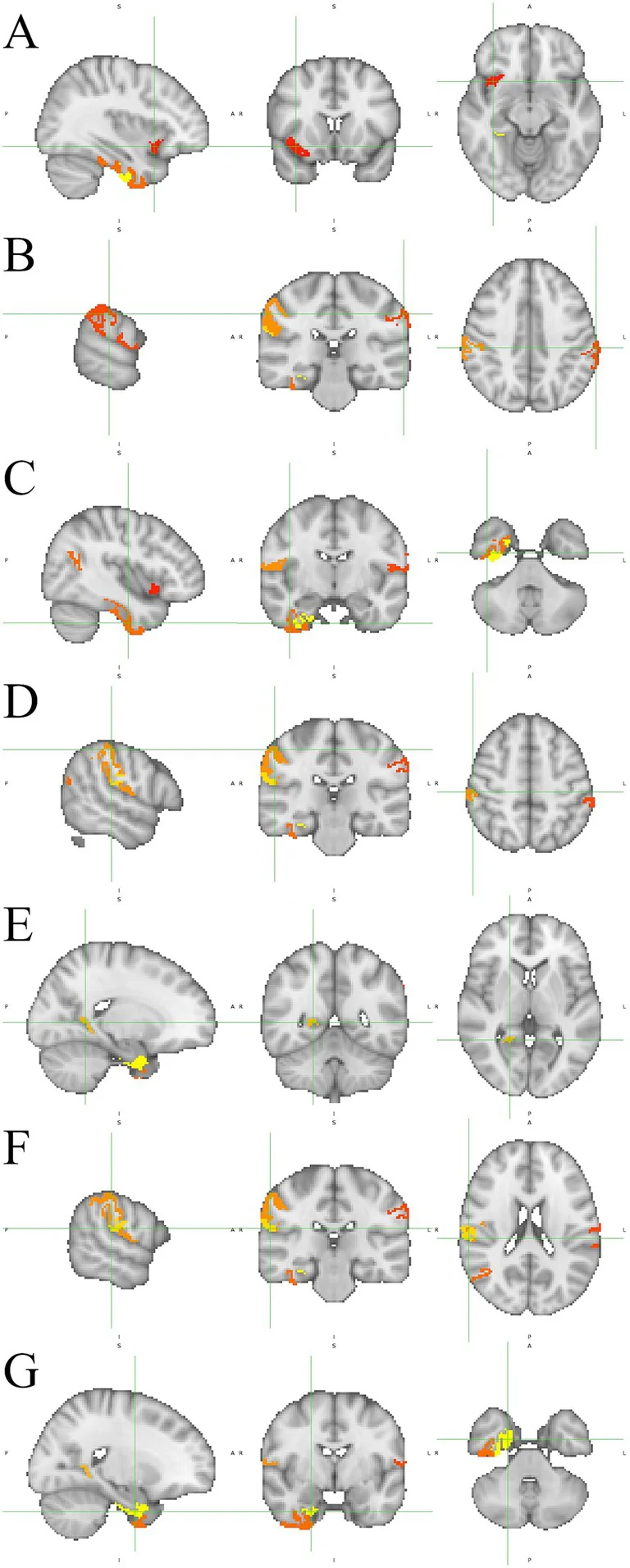
Networks with the highest SHAP average SHAP rankings across the folds (identified in Table 5) depicted over the ICBM152 brain template. Networks that contribute positively to mentalizing (MNT) are in the red-yellow range. Colors closer to red are associated with higher importance. Each pane consists of sagittal, coronal, and axial views of **(A)** Network 335 (*x* = 36, *y* = 14, *z* = −12); **(B)** Network 327 (*x* = 77, *y* = 49, *z* = 56); **(C)** Network 301 (*x* = 42, *y* = −10, *z* = −40); **(D)** Network 280 (*x* = 56, *y* = −26, *z* = 50); **(E)** Network 321 (*x* = 20, *y* = −50, *z* = 4); **(F)** Network 100 (*x* = 60, *y* = −26, *z* = 20); **(G)** Network 292 (*x* = 22, *y* = −4, *z* = −36). MNI152 standard space. Radiological convention. A: Anterior; I: Inferior; L: Left; P: Posterior; R: Right; S: Superior.

**Figure 6:**
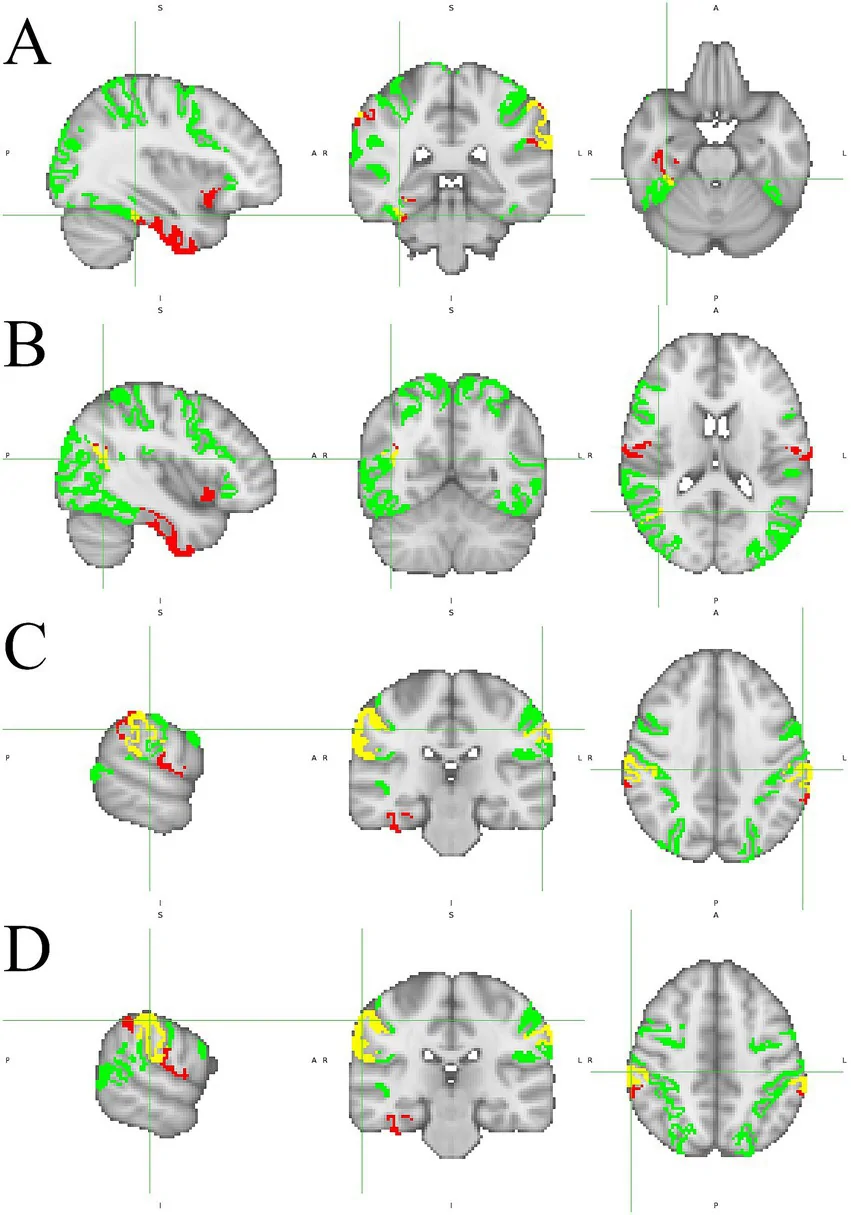
Networks identified as consistently contributing positively to mentalizing across folds overlapped with the GLM-based statistical parametric map obtained from (Dureux et al., 2023). The GLM-based map is masked with the MMP1.0 atlas to remove activations in voxels that fall outside of what is seen by the model. Regions that belong only to the identified networks are in red, areas that are present only in the literature are in green, and regions that are present in both (overlap) are in yellow. Each pane consists of sagittal, coronal, and axial views of **(A)** x = 36, y = −36, z = −24; **(B)** x = 42, y = −58, z = 16; **(C)** x = −62, y = −26, z = 38; **(D)** x = 62, y = −26, z = 46. MNI152 standard space. Radiological convention. A: anterior; I: inferior; L: left; P: posterior; R: right; S: superior.

### 3.4 Identification of regions within networks

Cohen’s d is computed across the regions of each retained multi-region network on the mnt instances of the pooled sample (Table 6); networks 327 and 292 comprise a single region and are not included. These values rank the regions within each network by relative signal magnitude during mentalizing, indicating which regions carry more signal than others in the same network, and are not intended to quantify absolute contributions.

**Table 6:** Cohen’s D values for the mentalizing instances across regions within each of the selected networks.

Networks	Cohen’s D mentalizing instances				
335	Region ID	302	335		
	302	0	0.017		
	335	−0.017	0		
301	Region ID	301	302		
	301	0	−0.140		
	302	0.140	0		
280	Region ID	280	296	328	
	280	0	−0.147	0.047	
	296	0.147	0	0.201	
	328	−0.047	−0.201	0	
321	Region ID	302	315	321	352
	302	0	0.186	0.347	0.043
	315	−0.186	0	0.142	−0.141
	321	−0.347	−0.142	0	−0.296
	352	−0.043	0.141	0.296	0
100	Region ID	100	116	148	
	100	0	−0.107	0.001	
	116	0.107	0	0.112	
	148	−0.001	−0.112	0	

A value closer to 0 indicates the regions have closer average signal magnitude in these instances. A higher absolute value (more distant from 0) indicates their signals are farther apart.

In network 335, regions 302 (Perirhinal/Ectorhinal Cortex R) and 335 (ParaHippocampal Area 2 R) have near-identical magnitudes (d = 0.017). In network 301, region 302 exceeds region 301 (ProStriate Area R) modestly (d = 0.14). In network 280, region 296 (Area PFt R) has the highest magnitude, followed by regions 280 (Area OP4-PV R) and 328 (Area PF Complex R), which are close to one another. In network 321, region 302 has the highest magnitude and region 321 (TPO Junction 3 R) the lowest, with regions 352 (Area TG ventral R) and 315 (Area TF R) intermediate; this network shows the largest within-network differentiation (d up to 0.35). In network 100, region 116 (Area PFt L) has the highest magnitude, followed by regions 100 (Area OP4-PV L) and 148 (Area PF Complex L), which are near-identical. Region 302 recurs across networks 335, 301, and 321 and is consistently among the higher-magnitude regions, suggesting a shared medial-temporal contribution. The complete 360 × 360 Cohen’s d matrix is provided in Supplementary Table 4.

These region-level results also permit a direct comparison with the baseline SHAP analysis, which ranks individual regions rather than structurally connected networks. Despite the two models reaching the same retrained accuracy on the shared fold (84.7%; Tables 1, 2), their rankings do not coincide. None of the baseline’s most important regions (ranks 1–16), belongs to any retained network. Several network constituents are nonetheless strong individual discriminators in the baseline (regions 296, 328, 116, 335, and 301, ranked 17th, 23rd, 32nd, 34th, and 42nd), so the two analyses partially agree on those regions. The clearest divergence is region 302, the only region shared across networks (335, 301, and 321) and consistently among their higher-magnitude regions, yet ranked only 149th in the baseline: the region that ties the mentalizing-positive networks together is individually unremarkable at the region level. Only one constituent region (315) is pruned in the baseline.

## 4 Discussion

This study introduces a structurally informed message-passing framework integrated into a shallow neural network classifier. The primary finding is that network-level modeling achieves performance comparable to a region-level baseline while substantially improving biological interpretability. On the partition shared with the baseline (the first cross-validation fold), the initial global accuracy is marginally lower in the proposed model (87.1% vs. 88.2%), yet both converge to the same retrained accuracy (84.7%); across all three folds the proposed model maintains comparable retrained accuracy (mean 85.1%), with the pruning-then-retraining recovery reproduced in every fold. These results demonstrate that incorporating structural connectivity does not reduce predictive validity, while it shifts the interpretative focus from isolated brain regions toward biologically plausible networks, thereby avoiding the concerns raised by Wen et al. (2018) (balancing predictive performance with robustness in low-data regimes). In the same way, the proposed framework avoids the risk of capturing spurious or biologically implausible patterns when anatomical constraints are not considered.

Whereas the baseline model identifies individual regions contributing to classification, the proposed approach constrains the propagation of information according to diffusion-based structural connectivity. As a consequence, the explanatory units correspond to structurally grounded networks rather than isolated nodes. This perspective aligns with systems-level approaches in Neuroscience, which conceptualize cognitive processes as emergent properties of distributed interactions across large-scale brain systems. In particular, processes such as Theory of Mind (ToM) are widely understood to rely on coordinated activity across multiple cortical regions rather than on a single localized substrate. The consistently higher post-retraining accuracy for the mentalizing condition across folds (mean 90.2% versus 79.2% for the random condition) suggests that structurally organized network recruitment is especially informative for socially relevant cognitive processes. Because the present analysis targets the mentalizing stimulus, it retains the inputs that consistently contribute toward the mentalizing class across folds; the inputs that consistently contribute toward the random class are their symmetric counterpart and could be analyzed in the same manner, but are not the focus here. The seven networks carried forward are thus mentalizing-positive across all three folds by construction of the selection rather than as an emergent property.

The cross-validated explanations also reveal a distinction that a single data split would obscure. The magnitude of the inputs’ contributions is reproducible across folds (Kendall’s W = 0.61), indicating that the model consistently relies on the same inputs; the direction of those contributions, however, is far less stable, with the signed rankings showing no concordance beyond chance (W = 0.37) and only about a quarter of the commonly retained inputs preserving their sign across all three folds. In other words, which inputs matter is reproducible, but whether a given input favors the mentalizing or the random class often depends on the particular subjects in the fold. This cautions against treating the directional attributions of a single split as reliable, and motivates the sign-consistency criterion adopted here: restricting the analysis to inputs whose direction is preserved across folds isolates the seven networks that can be interpreted as robustly mentalizing-related, the consistently random-positive inputs forming a symmetric counterpart outside the present focus. The stability of these explanations is therefore a property established by the cross-validation rather than assumed from a single fit.

The relatively small sample size used in this study reflects realistic constraints associated with task-based fMRI acquisition, where collecting large cohorts with complex paradigms can be challenging. The fact that the proposed model demonstrates stable convergence and interpretable results under these conditions suggests that the framework is well-suited for typical neuroimaging studies. The cross-validation further indicates that the identified networks are stable across resampling of the cohort; nevertheless, evaluating generalizability in larger and independent datasets remains an important direction for future work. Biological plausibility constitutes a central contribution of the proposed framework. Structurally constrained message-passing restricts the hypothesis space to anatomically feasible configurations, thereby reducing the likelihood that the classifier exploits spurious correlations unrelated to known brain organization. The inclusion of a diagonal term ensures that intra-regional contributions are preserved during signal propagation, while degree normalization mitigates signal dilution effects that could arise when nodes with many connections aggregate information from both informative and non-informative regions. Together, these mechanisms ensure that both local and distributed contributions to the neural signal are meaningfully represented.

Importantly, the spatial distribution of the networks identified as most relevant for classification shows some overlap along with a high number of adjacent regions to those previously implicated in ToM processing. In particular, the seven networks are concentrated in the temporo-parietal junction and inferior parietal cortex (the temporo-parieto-occipital junction and the PF and PFt complexes), the medial temporal lobe (parahippocampal and perirhinal/entorhinal cortices), and the insular and posterior cingulate cortices. These regions are repeatedly reported as components of the ToM and broader social-cognition network in prior studies (temporo-parietal junction: Lavoie et al., 2016; Ogawa and Matsuyama, 2023; Schurz et al., 2014; Young et al., 2010; parahippocampal/medial temporal: Qin et al., 2023). The correspondence between the networks highlighted by the present framework and regions consistently reported in the literature supports the biological relevance of the proposed method, and is shown in Figure 5 of the results. The overlap analysis also exposes a key limitation of conventional GLM-based approaches. A substantial proportion of the voxels identified as associated with the mentalizing condition are located in white-matter regions. From a neurophysiological perspective, this is unlikely to reflect task-related activation. The BOLD signal primarily originates from metabolic demand, which is concentrated in gray matter. In contrast, white matter is composed mainly of myelinated axonal fibers, exhibits lower metabolic activity, reduced vascular density, and weaker neurovascular coupling. Consequently, meaningful BOLD signal changes are not expected in these regions.

The presence of apparent white matter activations in GLM results is therefore most plausibly explained by methodological factors, particularly spatial smoothing. While smoothing is commonly applied to improve the signal-to-noise ratio and accommodate inter-subject variability, it also introduces partial-volume effects, effectively blurring signals across tissue boundaries. As a result, strong gray-matter activations can “leak” into adjacent white-matter voxels, leading to biologically implausible findings.

As reported in the Results, the network-level and region-level SHAP analyses give genuinely different accounts of the same classification: the baseline’s strongest individual regions (125, 145, 290, 80) are absent from the networks, whereas region 302, individually unremarkable (149th), becomes the hub shared across three mentalizing-positive networks. This suggests that structural connectivity redistributes importance rather than reproducing it, so that a region’s relevance at the network level depends on its participation in structurally coherent subnetworks and not on its individual discriminative power alone. In other words, network-level organization can capture distributed patterns of activity that are not apparent when features are evaluated independently.

This observation suggests that groups of regions with moderate individual importance may collectively provide stronger information about the distinction between mentalizing and random processes than any single region alone. In other words, network-level organization may capture distributed patterns of activity that are not apparent when features are evaluated independently. Still, this does not fully explain why most of the top-ten regions identified in the previous region-based analysis do not reappear in the most relevant networks. Several mechanisms may explain why certain regions gain prominence in the network-based analysis. One possibility relates to signal dilution: regions that were previously highly informative at the regional level may be structurally connected to many regions that are not directly involved in the task. When signals are aggregated through message-passing, the relevant activity may be partially diluted by baseline signals and noise from these additional regions.

Another possible explanation concerns the role of the hidden layer in the classifier. During training, the hidden nodes may integrate information from multiple structurally defined subnetworks to form higher-level representations that are more informative for classification. In this scenario, the importance of individual regions would depend not only on their own signal but also on how they interact with other regions within the learned representations. A detailed investigation of the signal time series after the message-passing step, as well as an analysis of the hidden-layer activations, would be required to evaluate these hypotheses.

The analysis of Cohen’s D values provides complementary insights into the structure of the network signals. Although these values primarily capture relative differences in signal magnitude and, therefore, cannot definitively determine whether specific regions are completely uninvolved in the task, several patterns nevertheless emerge. The effect sizes are modest and the within-network separations small, consistent with regions belonging to the same structurally defined network carrying broadly comparable signal during mentalizing. One regional pattern is nonetheless notable: region 302 (perirhinal/ectorhinal cortex) recurs across networks 335, 301, and 321 and is consistently among the higher-magnitude regions, pointing to a shared medial-temporal contribution. Paired with the SHAP analysis, this ranking extends the network-level explanation to the level of constituent regions, identifying which regions contribute most to each network’s signal without claiming that the remaining regions are uninvolved. One possible interpretation is that the larger effect sizes reflect heterogeneous activation within the networks: some regions may be selectively recruited during mentalizing, while others remain closer to baseline. Such variability would lead to larger differences in signal magnitude across regions within the same network. If confirmed, this pattern would suggest the existence of structurally constrained, yet functionally selective, subnetworks involved in mentalizing. However, this hypothesis requires further validation through analyses of absolute signal magnitudes rather than relying on relative measures alone. Even so, the current analysis already allows ranking regions within each network according to their expected contribution to the model’s classification decisions.

Clinically, the proposed framework may be particularly relevant to the study of neurological and psychiatric disorders, which are increasingly conceptualized as disorders of network connectivity. In conditions such as Alzheimer’s disease and Mild Cognitive Impairment, large-scale connectivity alterations often precede overt structural atrophy. Similarly, disorders including Attention Deficit Hyperactivity Disorder, Autism Spectrum Disorder, Bipolar Disorder, and Schizophrenia are increasingly described as dysconnectivity syndromes (Du et al., 2018). In such contexts, analytical frameworks that explicitly model interactions among structurally connected regions may yield more informative biomarkers than approaches that treat brain regions in isolation. By integrating anatomical constraints with interpretable machine learning methods, the present approach therefore offers a promising direction for studying both normative cognitive processes and network-level pathology.

An additional extension of the present framework concerns identifying regions that are active under both experimental conditions. A key limitation of classifier explanation methods, such as SHAP as employed in this study, is that they highlight features that differentiate between classes. Because the classifier is optimized to minimize prediction error, features that are similarly active in both conditions will tend to receive low importance scores. A similar limitation exists in traditional statistical analyses based on contrasts between conditions. In such approaches, regions engaged in both processes may be overlooked, even if they are functionally relevant to the task. When studying complex cognitive functions such as mentalizing, however, it is often desirable to identify all regions involved in the process rather than only those that differentiate it from a control condition. In the present framework, such regions may be detected by combining SHAP values with measures of signal magnitude. Regions that are highly active but show low SHAP importance may represent task-relevant components common to both conditions, whereas regions with both low SHAP values and low signal magnitude are more likely to be truly irrelevant to the task.

The adjacency matrix is pivotal within the method proposed here. The adjacency matrix must closely represent the biological network, internalizing in its parameters the tracts and fascicles that connect brain regions, faithfully capturing its small-world structure. This study integrates several works that used anatomy-driven deterministic tractography on diffusion-weighted images from 10 subjects (Baker et al., 2018). Human brain variability within normality may not have been fully accounted for, and population-based adjacency matrices may be designed, yielding different results. Structural impairments may also be modeled by the adjacency matrix. All these branches represent avenues for future work and comparison with the present results and interpretation.

In summary, the present study demonstrates that incorporating structurally constrained message-passing into an SNN classifier enables biologically accurate and interpretable brain decoding without compromising classification performance. By constraining information flow according to anatomical connectivity, the proposed framework shifts interpretation from isolated regions toward distributed brain networks that more closely reflect the organization of cognitive processes described in Neuroscience. The results show that network-level modeling achieves performance comparable to region-based approaches while highlighting subnetworks consistent with prior literature on Theory of Mind. Moreover, the combination of structural constraints and explainability methods such as SHAP enables the identification of networks and constituent regions that contribute to classification, providing insights into both differentiating and potentially shared components of task-related brain activity. Beyond the present paradigm, this framework offers a promising avenue for improving the interpretability of machine learning models applied to fMRI data, particularly in the study of cognitive processes and disorders characterized by alterations in large-scale brain connectivity.

## Data Availability

Publicly available datasets were analyzed in this study. This data can be found at: HCP-Young Adult 2025 at https://balsa.wustl.edu/project?project=HCP_YA.

## Footnotes

1 HCP Young Adult; https://www.humanconnectome.org/study/hcp-young-adult; accessed on 16 September 2024

2 https://github.com/shap/shap; accessed on 06 February 2026

3 https://pytorch.org/; accessed on 06 February 2026

## References

[ref1] Adolphs R. (2001). The neurobiology of social cognition. Curr. Opin. Neurobiol. 11, 231–239. doi: 10.1016/S0959-4388(00)00202-6, 11301245

[ref2] Baker C. M. Burks J. D. Briggs R. G. Conner A. K. Glenn C. A. Sali G. . (2018). A connectomic atlas of the human cerebrum-chapter 1: introduction, methods, and significance. Oper. Neurosurg. 15, S1–S9. doi: 10.1093/ons/opy253 PMC6887907 30260422

[ref3] Baliga V. B. Armstrong M. S. Press E. R. Bonnet-Lebrun A. S. Sciaini M. (2023). *Pathviewr: Wrangle, Analyze, and Visualize Animal Movement Data (v. 1.1.7)*. Available online at: https://cran.r-project.org/package=pathviewr.

[ref4] Bassett D. S. Sporns O. (2017). Network neuroscience. Nat. Neurosci. 20, 353–364. doi: 10.1038/nn.4502, 28230844 PMC5485642

[ref5] Briggs R. G. Conner A. K. Baker C. M. Burks J. D. Glenn C. A. Sali G. . (2018a). A connectomic atlas of the human cerebrum-chapter 18: the connectional anatomy of human brain networks. Oper. Neurosurg. 15, S470–S480. doi: 10.1093/ons/opy272 PMC6890524 30260432

[ref6] Briggs R. G. Conner A. K. Rahimi M. Sali G. Baker C. M. Burks J. D. . (2018b). A connectomic atlas of the human cerebrum-chapter 14: Tractographic description of the frontal aslant tract. Oper. Neurosurg. 15, S444–S449. doi: 10.1093/ons/opy268, 30260440 PMC6890526

[ref7] Briggs R. G. Conner A. K. Sali G. Rahimi M. Baker C. M. Burks J. D. . (2018c). A connectomic atlas of the human cerebrum-chapter 16: Tractographic description of the vertical occipital fasciculus. Oper. Neurosurg. 15, S456–S461. doi: 10.1093/ons/opy270, 30260427 PMC6890522

[ref8] Briggs R. G. Conner A. K. Sali G. Rahimi M. Baker C. M. Burks J. D. . (2018d). A connectomic atlas of the human cerebrum-chapter 17: tractographic description of the cingulum. Oper. Neurosurg. 15, S462–S469. doi: 10.1093/ons/opy271, 30260430 PMC6890528

[ref9] Briggs R. G. Rahimi M. Conner A. K. Sali G. Baker C. M. Burks J. D. . (2018e). A connectomic atlas of the human cerebrum-chapter 15: tractographic description of the uncinate fasciculus. Oper. Neurosurg. 15, S450–S455. doi: 10.1093/ons/opy269, 30260439 PMC6890529

[ref10] Call J. Tomasello M. (2008). Does the chimpanzee have a theory of mind? 30 years later. Trends Cogn. Sci. 12, 187–192. doi: 10.1016/j.tics.2008.02.010, 18424224

[ref11] Chauhan A. S. Singh R. Priyadarshi N. Twala B. Suthar S. Swami S. (2024). Unleashing the power of advanced technologies for revolutionary medical imaging: pioneering the healthcare frontier with artificial intelligence. Discov. Artif. Intell. 4:58. doi: 10.1007/s44163-024-00161-0

[ref12] Conner A. K. Briggs R. G. Rahimi M. Sali G. Baker C. M. Burks J. D. . (2018a). A connectomic atlas of the human cerebrum-chapter 10: tractographic description of the superior longitudinal fasciculus. Oper. Neurosurg. 15, S407–S422. doi: 10.1093/ons/opy264, 30260421 PMC6887962

[ref13] Conner A. K. Briggs R. G. Rahimi M. Sali G. Baker C. M. Burks J. D. . (2018b). A connectomic atlas of the human cerebrum-chapter 12: tractographic description of the middle longitudinal fasciculus. Oper. Neurosurg. 15, S429–S435. doi: 10.1093/ons/opy266, 30260450 PMC6887674

[ref14] Conner A. K. Briggs R. G. Sali G. Rahimi M. Baker C. M. Burks J. D. . (2018c). A connectomic atlas of the human cerebrum-chapter 13: tractographic description of the inferior fronto-occipital fasciculus. Oper. Neurosurg. 15, S436–S443. doi: 10.1093/ons/opy267, 30260438 PMC6890527

[ref15] Decety J. Keenan J. P. (2006). Social neuroscience: a new journal. Soc. Neurosci. 1, 1–4. doi: 10.1080/17470910600683549, 18633771

[ref16] Doran D. Schulz S. Besold T. R. (2018). What does Explainable AI Really mean? A New Conceptualization of Perspectives. *Proceedings of the 1st International Workshop on Comprehensibility and Explanation in AI and ML, CEX 2017*. CEUR Workshop Proceedings.

[ref17] Du Y. Fu Z. Calhoun V. D. (2018). Classification and prediction of brain disorders using functional connectivity: promising but challenging. Front. Neurosci. 12:525. doi: 10.3389/fnins.2018.00525 PMC6088208 30127711

[ref18] Dureux A. Zanini A. Selvanayagam J. Menon R. S. Everling S. (2023). Gaze patterns and brain activations in humans and marmosets in the Frith-Happé theory-of-mind animation task. eLife 12:e86327. doi: 10.7554/eLife.86327, 37449983 PMC10435231

[ref19] Elam J. S. Glasser M. F. Harms M. P. Sotiropoulos S. N. Andersson J. L. R. Burgess G. C. . (2021). The human connectome project: a retrospective. NeuroImage 244:118543. doi: 10.1016/j.neuroimage.2021.118543, 34508893 PMC9387634

[ref20] Farahani F. V. Fiok K. Lahijanian B. Karwowski W. Douglas P. K. (2022). Explainable AI: a review of applications to neuroimaging data [systematic review]. Front. Neurosci. 16:906290. doi: 10.3389/fnins.2022.906290 PMC9793854 36583102

[ref22] Glasser M. F. Coalson T. S. Robinson E. C. Hacker C. D. Harwell J. Yacoub E. . (2016). A multi-modal parcellation of human cerebral cortex. Nature 536, 171–178. doi: 10.1038/nature18933, 27437579 PMC4990127

[ref23] Haykin S. (2009). Neural Networks and Learning Machines. 3rd Edn Hoboken, NJ: Prentice Hall.

[ref24] Heider F. Simmel M. (1944). An experimental study of apparent behavior. Am. J. Psychol. 57, 243–259. doi: 10.2307/1416950

[ref25] Lavoie M.-A. Vistoli D. Sutliff S. Jackson P. L. Achim A. M. (2016). Social representations and contextual adjustments as two distinct components of the theory of mind brain network: evidence from the REMICS task. Cortex 81, 176–191. doi: 10.1016/j.cortex.2016.04.017, 27236373

[ref26] Limas M. C. Meré J. B. O. Marcos A. G. Ascacibar F. J. M. Espinoza A. V. P. Elías F. A. . (2014). *AMORE: A MORE Flexible Neural Network Package (0.2–15)*. In (Version 0.2-15) Available online at: http://cran.nexr.com/web/packages/AMORE/index.html

[ref27] Lundberg S. M. Lee S. I. (2017). *A Unified Approach to Interpreting model Predictions Advances in Neural Information Processing Systems 30 (NIPS 2017)*, Long Beach (CA), USA. Available online at: https://proceedings.neurips.cc/paper/2017/file/8a20a8621978632d76c43dfd28b67767-Paper.pdf.

[ref28] Marques dos Santos J. D. Marques dos Santos J. P. (2022). “Towards XAI: Interpretable Shallow Neural Network Used to Model HCP’s fMRI Motor Paradigm Data,” in Bioinformatics and Biomedical Engineering. Lecture Notes in Computer Science, eds. Rojas I. Valenzuela O. Rojas F. Herrera L. J. Ortuño F., vol. 13347 (Berlin: Springer International Publishing), 260–274.

[ref29] Marques dos Santos J. D. Marques dos Santos J. P. (2023). “Path Weights Analyses in a Shallow Neural Network to Reach Explainable Artificial Intelligence (XAI) of fMRI Data,” in Machine Learning, Optimization, and Data Science. Lecture Notes in Computer Science, eds. Nicosia G. Ojha V. La Malfa E. La Malfa G. Pardalos P. Di Fatta G., vol. 13811 (Berlin: Springer International Publishing), 417–431.

[ref30] Marques dos Santos J. D. Reis L. P. Marques dos Santos J. P. (2025). Decoding mental states in social cognition: insights from explainable artificial intelligence on HCP fMRI data. Mach. Learn. Knowl. Extr. 7:17. doi: 10.3390/make7010017

[ref31] Marques dos Santos J. P. Marques dos Santos J. D. (2024). XAI (explainable artificial intelligence) in neuromarketing / consumer neuroscience: an fMRI study on brand perception. Front. Hum. Neurosci. 18:1305164. doi: 10.3389/fnhum.2024.1305164 PMC10995351 38584851

[ref32] Mohammadi H. Karwowski W. (2025). Graph neural networks in brain connectivity studies: methods, challenges, and future directions. Brain Sci. 15:17. doi: 10.3390/brainsci15010017, 39851385 PMC11763835

[ref33] Norman G. J. Cacioppo J. T. Berntson G. G. (2010). Social neuroscience. Wiley Interdiscip. Rev. Cogn. Sci. 1, 60–68. doi: 10.1002/wcs.29, 26272839

[ref34] Ogawa K. Matsuyama Y. (2023). Heterogeneity of social cognition between visual perspective-taking and theory of mind in the temporo-parietal junction. Neurosci. Lett. 807:137267. doi: 10.1016/j.neulet.2023.137267, 37094640

[ref35] Otsuka Y. Osaka N. Ikeda T. Osaka M. (2009). Individual differences in the theory of mind and superior temporal sulcus. Neurosci. Lett. 463, 150–153. doi: 10.1016/j.neulet.2009.07.064, 19632299

[ref36] Pereira F. Mitchell T. M. Botvinick M. (2009). Machine learning classifiers and fMRI: a tutorial overview. NeuroImage 45, S199–S209. doi: 10.1016/j.neuroimage.2008.11.007, 19070668 PMC2892746

[ref37] Premack D. Woodruff G. (1978). Does the chimpanzee have a theory of mind? Behav. Brain Sci. 1, 515–526. doi: 10.1017/S0140525X00076512

[ref38] Qin X. Huang H. Liu Y. Zheng F. Zhou Y. Wang H. (2023). Increased functional connectivity involving the parahippocampal gyrus in patients with schizophrenia during theory of mind processing: a psychophysiological interaction study. Brain Sci. 13:692. doi: 10.3390/brainsci13040692, 37190657 PMC10136879

[ref39] Repple J. Gruber M. Mauritz M. de Lange S. C. Winter N. R. Opel N. . (2023). Shared and specific patterns of structural brain connectivity across affective and psychotic disorders. Biol. Psychiatry 93, 178–186. doi: 10.1016/j.biopsych.2022.05.031 36114041

[ref40] Roscher R. Bohn B. Duarte M. F. Garcke J. (2020). Explainable machine learning for scientific insights and discoveries. IEEE Access 8, 42200–42216. doi: 10.1109/ACCESS.2020.2976199

[ref41] Sali G. Briggs R. G. Conner A. K. Rahimi M. Baker C. M. Burks J. D. . (2018). A connectomic atlas of the human cerebrum-chapter 11: tractographic description of the inferior longitudinal fasciculus. Oper. Neurosurg. (Hagerstown, Md.) 15, S423–S428. doi: 10.1093/ons/opy265, 30260434 PMC6887999

[ref42] Saxe R. (2006). “Four Brain Regions for one Theory of Mind?” in Social Neuroscience: People Thinking about Thinking People, eds. Cacioppo J. T. Visser P. S. Pickett C. L. (Cambridge, MA: MIT Press), 83–101.

[ref43] Schurz M. Radua J. Aichhorn M. Richlan F. Perner J. (2014). Fractionating theory of mind: a meta-analysis of functional brain imaging studies. Neurosci. Biobehav. Rev. 42, 9–34. doi: 10.1016/j.neubiorev.2014.01.009, 24486722

[ref44] Shamay-Tsoory S. G. Aharon-Peretz J. Perry D. (2009). Two systems for empathy: a double dissociation between emotional and cognitive empathy in inferior frontal gyrus versus ventromedial prefrontal lesions. Brain 132, 617–627. doi: 10.1093/brain/awn279, 18971202

[ref45] Shrikumar A. Greenside P. Kundaje A. (2017). *Learning Important Features through Propagating Activation Differences*. 34th International Conference on Machine Learning, PMLR.

[ref46] Sievers B. Thornton M. A. (2024). Deep social neuroscience: the promise and peril of using artificial neural networks to study the social brain. Soc. Cogn. Affect. Neurosci. 19:nsae014. doi: 10.1093/scan/nsae014, 38334747 PMC10880882

[ref47] Singer T. Tusche A. (2014). “Understanding Others: Brain Mechanisms of Theory of Mind and Empathy,” in Neuroeconomics: Decision Making and the Brain, eds. Glimcher P. W. Fehr E.. 2nd ed (New York: Academic Press), 513–532.

[ref49] Van Essen D. C. Glasser M. F. (2016). The human connectome project: progress and prospects. Cerebrum. 2016:cer-10-16.

[ref50] Van Essen D. C. Smith S. M. Barch D. M. Behrens T. E. J. Yacoub E. Ugurbil K. (2013). The WU-Minn human connectome project: an overview. NeuroImage 80, 62–79. doi: 10.1016/j.neuroimage.2013.05.041, 23684880 PMC3724347

[ref51] Van Mourik F. Jutte A. Berendse S. E. Bukhsh F. A. Ahmed F. (2024). Tertiary review on explainable artificial intelligence: where do we stand? Mach. Learn. Knowl. Extr. 6, 1997–2017. doi: 10.3390/make6030098

[ref52] Vilone G. Longo L. (2021). Classification of explainable artificial intelligence methods through their output formats. Mach. Learn. Knowl. Extr. 3, 615–661. doi: 10.3390/make3030032

[ref53] Wen D. Wei Z. Zhou Y. Li G. Zhang X. Han W. (2018). Deep learning methods to process fMRI data and their application in the diagnosis of cognitive impairment: a brief overview and our opinion. Front. Neuroinform. 12:23. doi: 10.3389/fninf.2018.00023 PMC5932168 29755334

[ref9001] Wu F. Zhang T. De Souza A. H. Fifty C. Yu T. Weinberger K. Q. (2019). Simplifying graph convolutional networks. arXiv (Cornell University). doi: 10.48550/arxiv.1902.07153

[ref54] Young L. Dodell-Feder D. Saxe R. (2010). What gets the attention of the temporo-parietal junction? An fMRI investigation of attention and theory of mind. Neuropsychologia 48, 2658–2664. doi: 10.1016/j.neuropsychologia.2010.05.012, 20470808

