# Supplementary material for: Structurally restricted message-passing within shallow architectures for explainable network-level brain decoding on small cohorts
Source: Front Behav Neurosci. 2026 Sep 23;20:1833995. doi: 10.3389/fnbeh.2026.1833995 (PMC13642823; doi:10.3389/fnbeh.2026.1833995)
Supplement: Supplementary file 1 [file Data_Sheet_1.PDF]

Supplementary Material

1 HCP MMP1.0 Regions

Supplementary Table 1 (supplementary tables are available in the Excel file "supplementary_tables" for better readability) is the complete list of all 360 regions used in the study, along with their ID, region abbreviation, region name, hemisphere, lobe, cortex ID, cortex, x, y, and z coordinates, and volume.

2 Connectivity Matrix

Supplementary Table 2 (supplementary tables are available in the Excel file "supplementary_tables" for better readability) is the connectivity matrix obtained from the literature after adding the diagonal term to account for intra-regional contributions during the message-passing.

3 SHAP

Supplementary Figure 1 depicts the full lists of the Shapley values for mentalizing and random conditions in the retrained network of fold 1. The networks (features) are ranked by their importance. Supplementary Figure 2 depicts the full lists of the Shapley values for mentalizing and random conditions in the retrained network of fold 2. The networks (features) are ranked by their importance. Supplementary Figure 3 depicts the full lists of the Shapley values for mentalizing and random conditions in the retrained network of fold 3. The networks (features) are ranked by their importance. The more important networks for the model's final prediction are plotted closer to the top.

Supplementary Table 3 (supplementary tables are available in the Excel file "supplementary_tables" for better readability) has the SHAP values and rankings per fold for the 93 inputs that survive pruning in all three folds. Since these SHAP values are computed using only mentalizing instances there is no longer a need to use absolute value and, thus, they're signed. A very negative value means high importance for the opposite class (random).

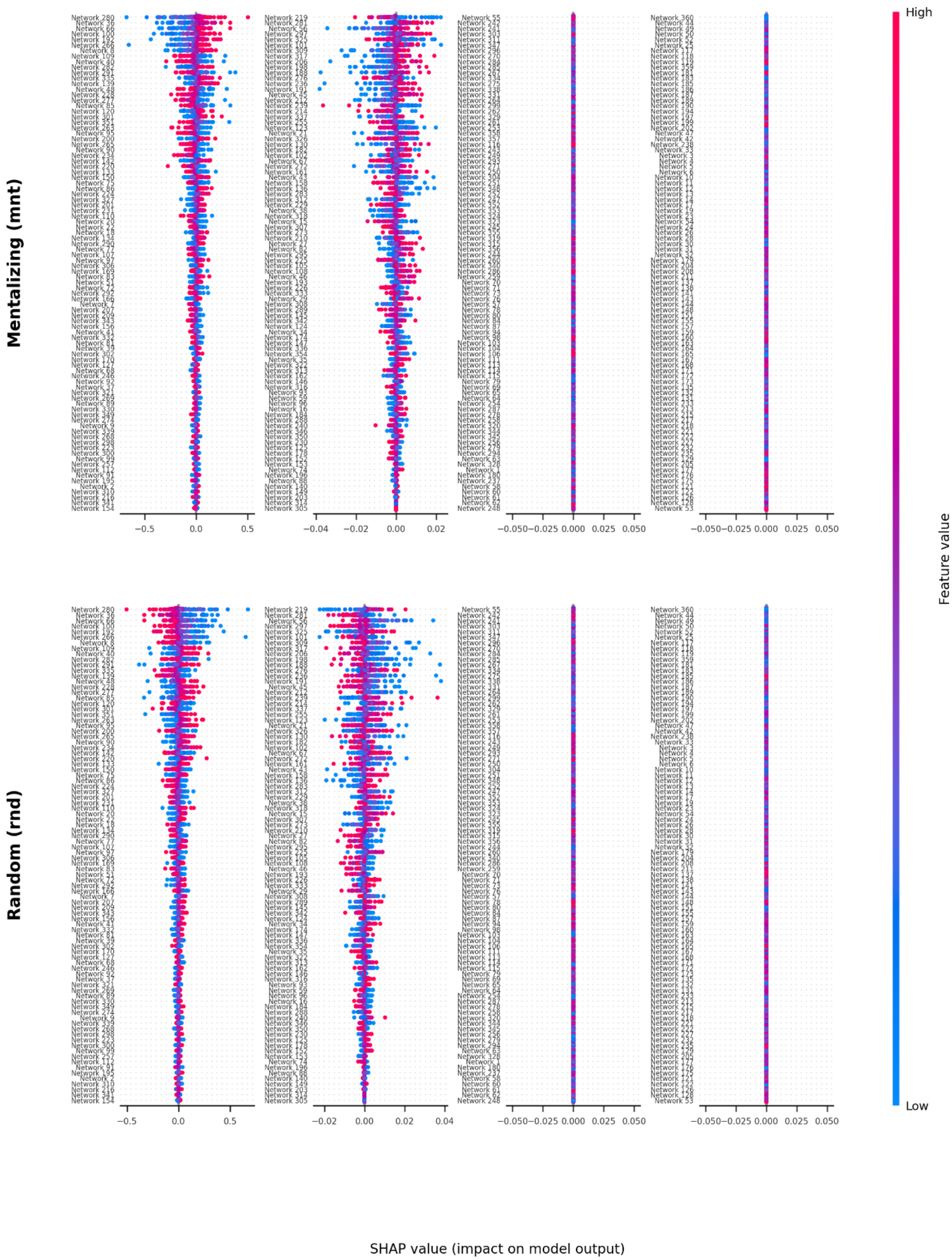

Supplementary Figure 1 Plot containing the distribution of SHAP values of the retrained network of fold 1 for all networks (inputs) for both category. Higher rank means more influence in the output computation either positively (red on the right), or negatively (blue on the right).

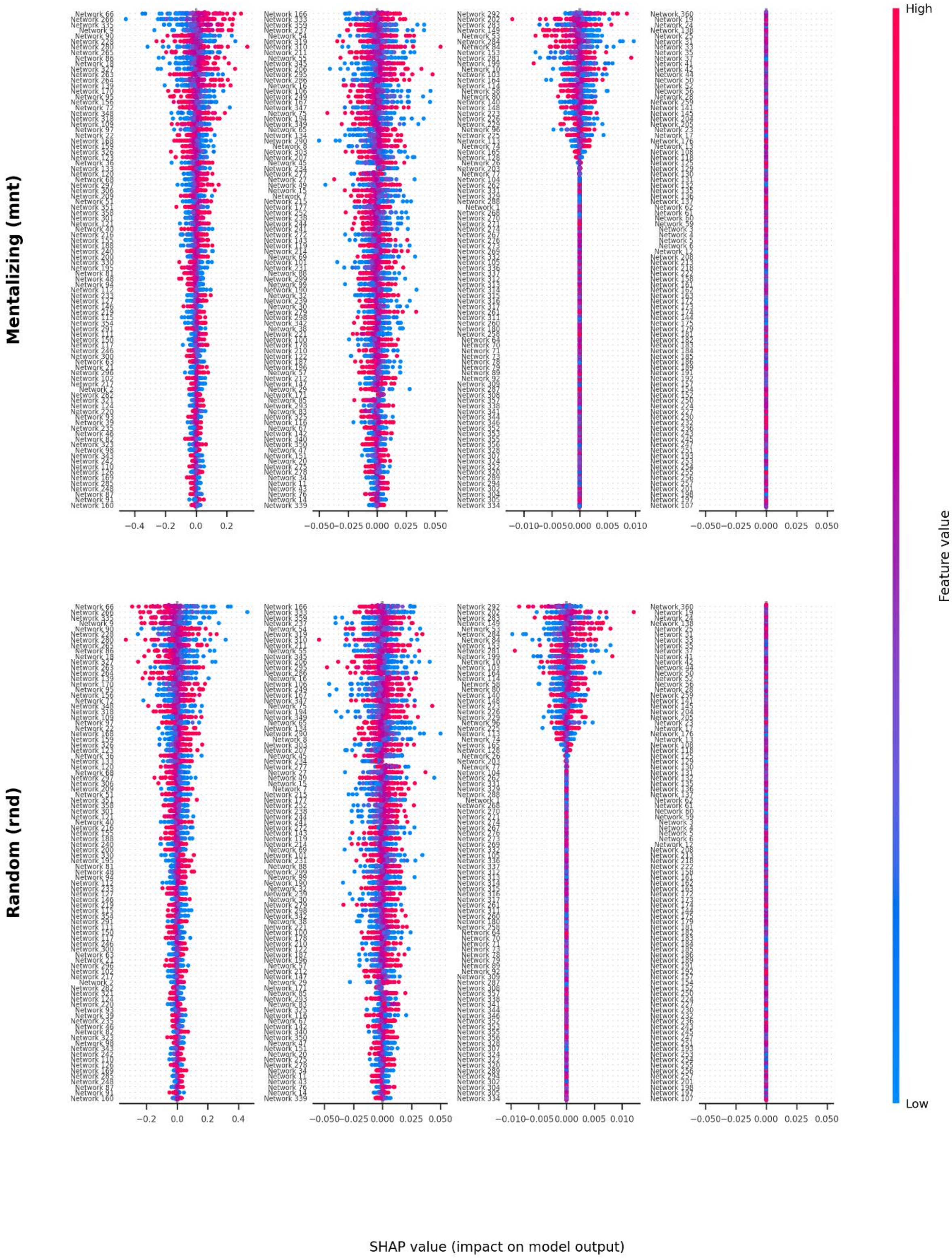

Supplementary Figure 2 Plot containing the distribution of SHAP values of the retrained network of fold 2 for all networks (inputs) for both category. Higher rank means more influence in the output computation either positively (red on the right), or negatively (blue on the right).

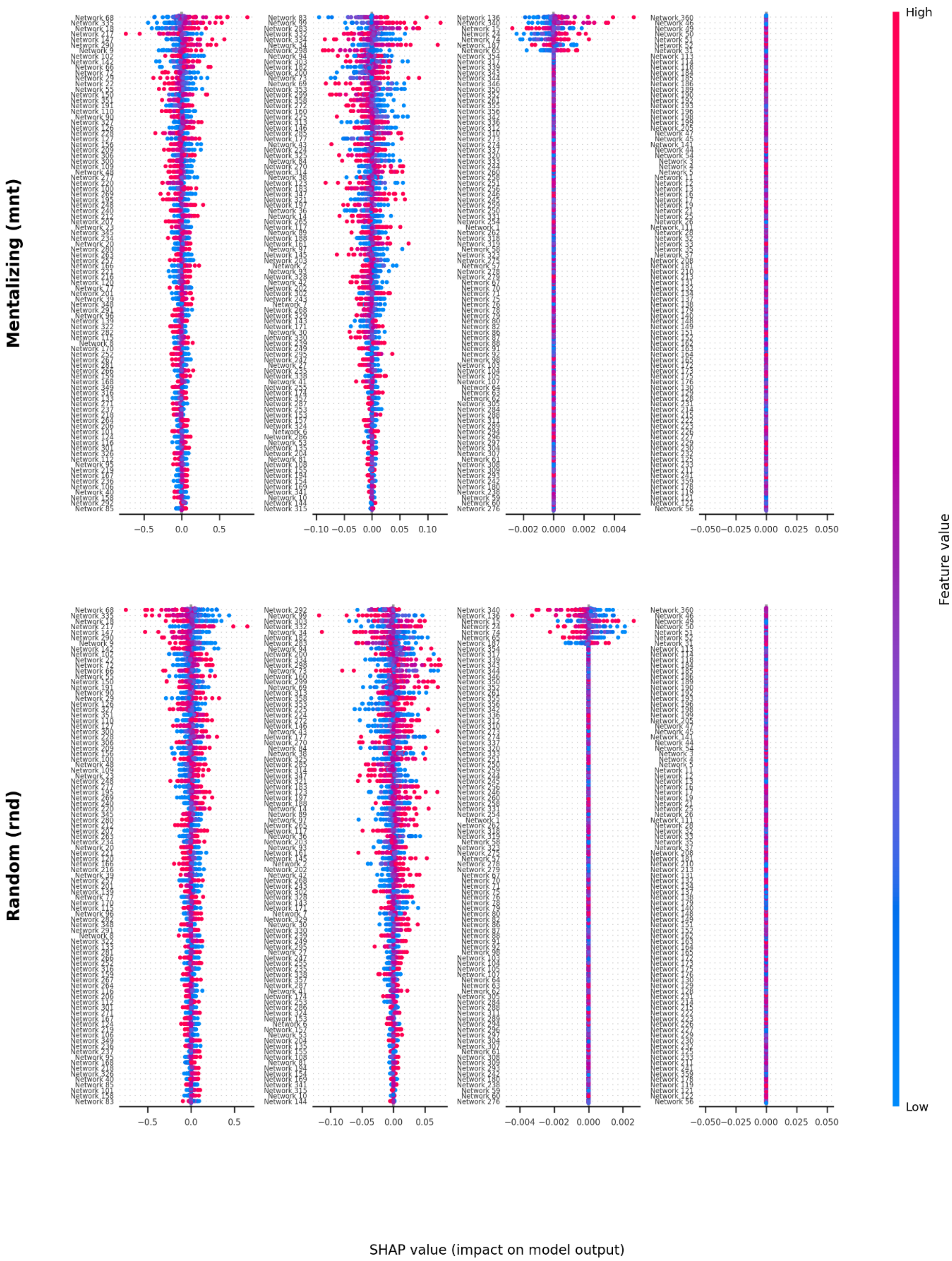

Supplementary Figure 3 Plot containing the distribution of SHAP values of the retrained network of fold 3 for all networks (inputs) for both category. Higher rank means more influence in the output computation either positively (red on the right), or negatively (blue on the right).

4 Cohen's D values

Supplementary Table 4 (supplementary tables are available in the Excel file "supplementary_tables" for better readability) is the complete matrix of Cohen's D values for all possible region pairs for the mentalizing instances. The Cohen's D values are computed by concatenating the regions signal matrix from each of the folds' test split, such that every subject is considered once and only once.
